# Epithelial-Myoepithelial Carcinoma of the Maxilla Arising From Minor Salivary Glands of Hard Palate: A Rare Case Report

**DOI:** 10.7759/cureus.45431

**Published:** 2023-09-17

**Authors:** Nour M Khattab, Aude Grand, Pierre Luc Descols, Jean-Jacques Brau, Odile Casiraghi, Pierre Khneisser, Ingrid Breuskin, Louis Maman, Ihsène Taihi

**Affiliations:** 1 Odontology, Health Faculty, University Paris Cité, Montrouge, FRA; 2 Oral Surgery, Assistance Publique-Hôpitaux de Paris (AP-HP) Rothschild Hospital, Paris, FRA; 3 Oral Surgery, Assistance Publique-Hôpitaux de Paris (AP-HP) Charles Foix Hospital, Ivry-sur-Seine, FRA; 4 Odontology and Maxillofacial Prosthesis Unit, Cervicofacial Cancerology, Gustave Roussy Cancer Center, Villejuif, FRA; 5 Pathology, Gustave Roussy Cancer Center, Villejuif, FRA; 6 Head and Neck Oncology, Gustave Roussy Cancer Center, Villejuif, FRA; 7 Laboratory of Orofacial Pathologies, Imaging, and Biotherapies, University Paris Cité, Montrouge, FRA

**Keywords:** salivary glands, humans, maxilla, maxillary sinus neoplasms, salivary gland neoplasms, mouth neoplasms

## Abstract

Epithelial-myoepithelial carcinoma is a rare malignant neoplasm of salivary glands. It is specifically found in the major salivary glands. The cases that emerge from minor salivary glands are rarely described. Histologically, it commonly exhibits a characteristic biphasic pattern consisting of epithelial and myoepithelial components. The histopathological resemblance to other benign and malignant neoplasms that also display myoepithelial characteristics makes the differential diagnosis challenging. Each differential diagnosis requires a very different management approach.

Considering the difficulties of anatomopathological diagnosis and the rarity of epithelial-myoepithelial carcinomas emerging from minor salivary glands, we report a rare epithelial-myoepithelial carcinoma case of minor salivary glands in a 58-year-old woman. She was referred for a palatal swelling, evolving for more than 35 years, and reported recent pain and nasal obstruction. The mucosal swelling was located in the left maxilla within the hard palate, of a 45-mm-long axis crossing the medial line and extending to the premaxilla, without cervical lymph node involvement. A computed tomography scan revealed a palatal lesion involving the left and the right maxilla. Furthermore, the superior alveolar process, both left and right maxillary sinuses, the nasal cavities, and the nasal septum were included in the lesion. The final diagnosis was difficult to confirm despite multiple biopsies and was determined only from the excised specimen.

The diagnosis of this tumor was challenging due to the clinical and histological similarities with other salivary tumors. The aim of this case report is to shed light on the distinctive features of these tumors and explore optimal screening and related management strategies.

## Introduction

Epithelial-myoepithelial carcinoma (EMC) is a rare malignant tumor of salivary glands. Its prevalence is estimated in recent studies from 0.4% to 1% of all salivary gland tumors [1,2]. The mean age of EMC diagnosis is around 60 years and females are slightly more affected [3,4]. EMC has a high recurrence rate of 35% to 40% [5]. In EMC, the mortality rate is low and that is why it is classified as a low-grade tumor [2,5]. The five and 10-year survival rates are high and can reach 94% and 90%, respectively [3]. This tumor is commonly found in the major salivary glands, including the parotid and submandibular glands [3,6]. The EMC cases that emerge from minor salivary glands are rarely described [3]. Histologically, the EMC commonly exhibits a characteristic biphasic pattern consisting of two distinct cell types that can vary in their relative proportions [2]. These two cell types consist of inner ductal epithelial cells that exhibit eosinophilic properties and outer myoepithelial cells that appear clear. Multiple histological variants of EMCs, like sebaceous, apocrine, and double-clear have also been described. In addition, high-grade EMCs were rarely described, and present an aggressive behavior with worse prognosis [3,7].

The histopathological resemblance of EMCs to other benign and malignant neoplasms that also display clear myoepithelial characteristics makes EMC differential diagnosis challenging [3]. Indeed, the histopathological differential diagnosis of EMC involves considering several other salivary gland neoplasms, such as pleomorphic adenoma (PA), myoepithelial carcinoma, and adenoid cystic carcinoma (ACC). ACC, classified as an intermediate to high-grade tumor according to the 2022 WHO classification, carries a poorer prognosis and requires a separate management approach compared to EMC [6,8].

Considering the challenges associated with anatomopathological diagnosis and the rarity of EMCs emerging from minor salivary glands, we report a rare clinical case of minor salivary gland EMC. We aim to shed light on the distinctive features of these tumors and explore optimal screening and related management strategies [2,3,6].

## Case presentation

**Figure 1:**
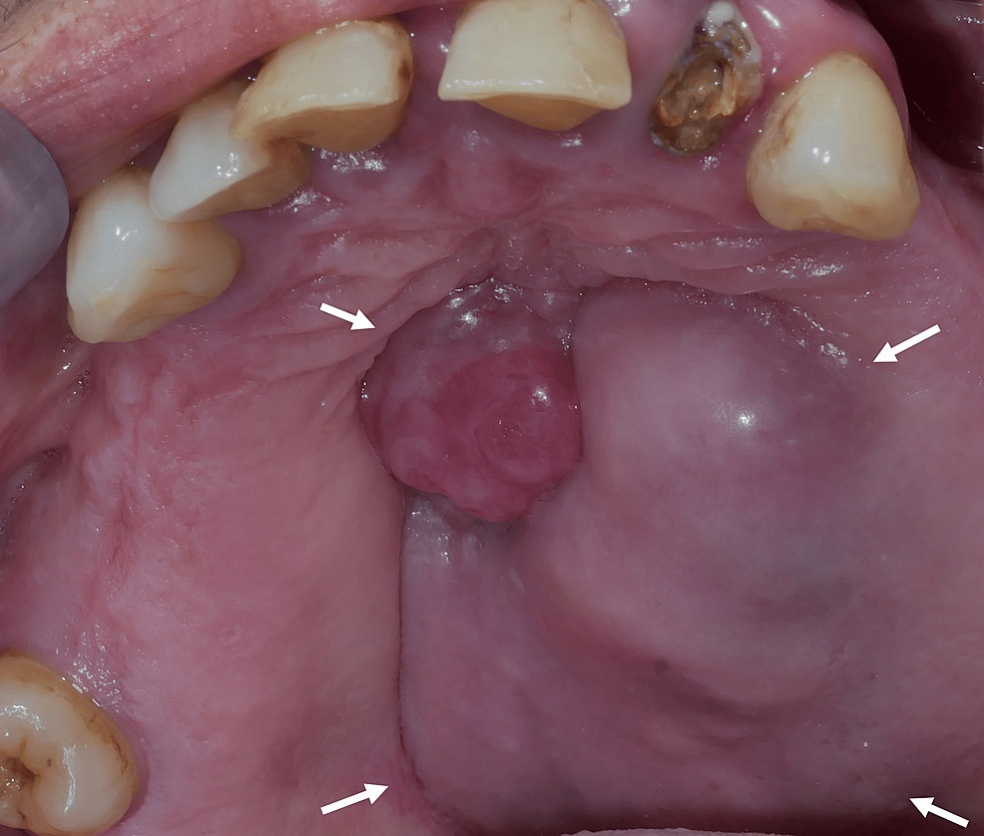
Clinical view Mucosal swelling of the left maxilla within the hard palate, of a 45-mm-long axis crossing the medial line and extending to the premaxilla, with a purplish-red color and polypoid outgrowth. (White arrows show the tumor’s limits.)

A 58-year-old woman was referred by a general dentist for a palatal swelling, slowly evolving and completely overlooked by the patient for more than 35 years. No prior biopsies or treatments have been provided. The patient reported occasional pain and nasal obstruction for three weeks. She was not undergoing any treatment for these complaints. She had high blood pressure treated with candesartan and indapamide, hypercholesterolemia treated with atorvastatin, and hypothyroidism treated with levothyroxine. The patient presented active smoking with 30 pack-years. Physical examination revealed a mucosal swelling of the left maxilla within the hard palate, of a 45-mm-long axis crossing the medial line and extending to the premaxilla. This palatal mucosa swelling had a purplish-red color with polypoid outgrowth. It was firm on palpation (Figure 1). The examination didn't show any cervical lymphadenopathies or signs of deterioration of the general status.

**Figure 2:**
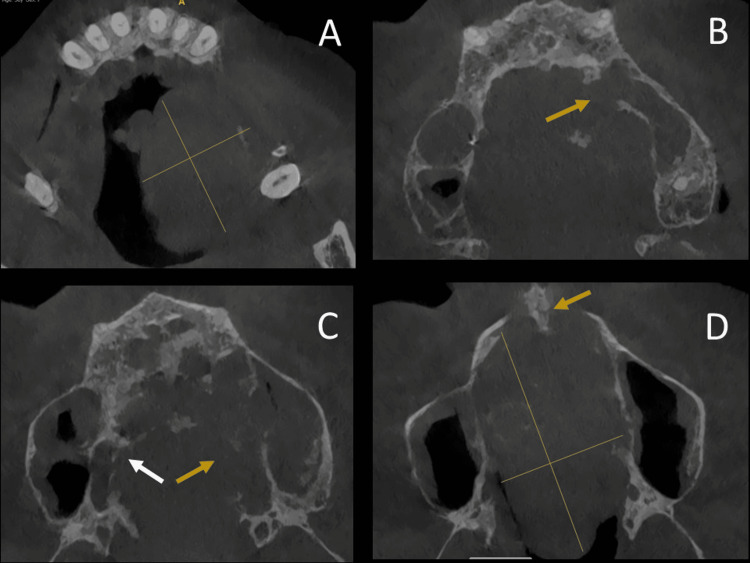
CT scan A: CT scan revealing the involvement of the superior left alveolar process. (The yellow cross shows the tumor’s extent.) B: CT scan revealing the involvement of the left and the right maxilla. (The yellow arrow shows the erosion of the left maxillary sinus medial wall.) C: CT scan revealing the involvement of the maxilla and both left and right maxillary sinuses. The lesion is crossing the midline. (The yellow and white arrows show the involvement of the left and right maxilla respectively.) D: CT scan revealing the involvement of the lower part of the nasal septum (as shown by the yellow arrow). (The yellow cross shows the tumor’s extent.) CT: computed tomography

Cone beam computed tomography (CBCT) and craniofacial computed tomography (CT) scan revealed a 45-mm-long axis, isodense lesion of the bony palate involving the left and the right maxilla, the superior left alveolar process, the nasal cavities, the lower part of the nasal septum and both left and right maxillary sinuses, with erosion of their medial wall. The lesion is rather lateralized to the left but extensively crosses the midline (Figure 2).

**Figure 3:**
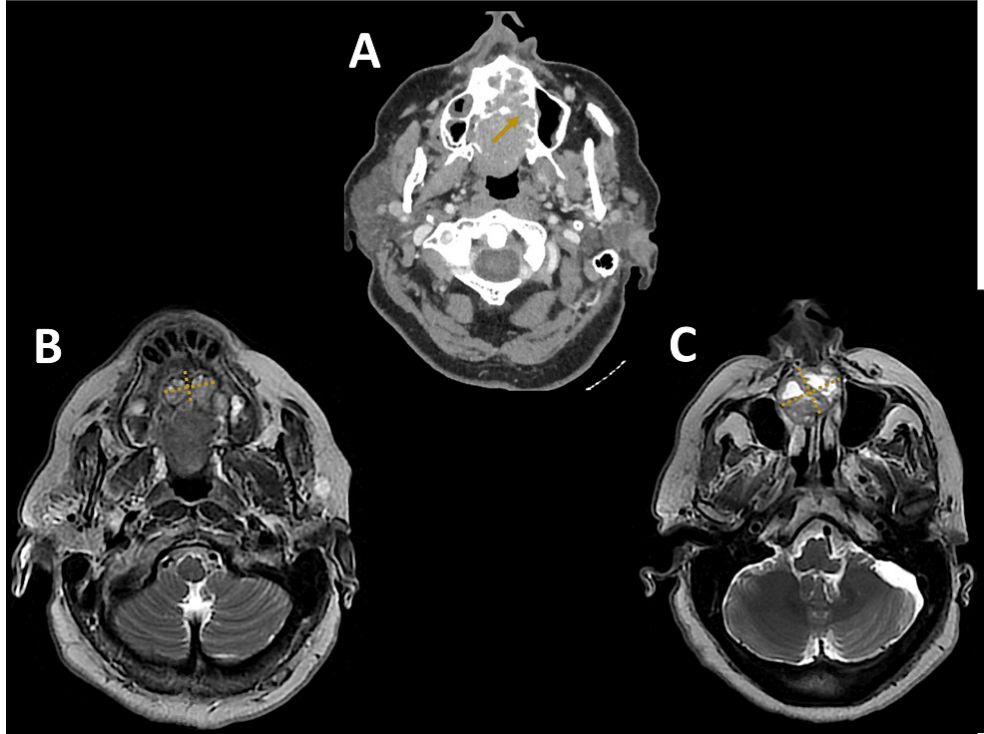
MRI A and B: T1 gadolinium craniofacial MRI shows a tumor taking up gadolinium heterogeneously, centered on the hard palate and extending posteriorly to the soft palate. (The yellow arrow shows bone invasion in the hard palate, and the yellow cross shows the tumor’s limits.) C: T1 gadolinium craniofacial MRI shows the extension of the lesion above and in front of the right and left nasal cavities and involvement of the septum and the lower part of the maxillary sinus. (The yellow cross shows the tumor’s limits). MRI: magnetic resonance imaging

T1 gadolinium craniofacial magnetic resonance imaging (MRI) showed a tumor taking up gadolinium heterogeneously. The lesion was located in the hard palate with posterior extension to the soft palate. It measured 56mm, 44mm, and 36mm in length, height, and width respectively. MRI showed also bone invasion in most of the hard palate, in the right and left nasal cavities, and in the inferior turbinates. The maxillary sinus was also involved with lysis of the uncinate process on the left (Figure 3).

**Figure 4:**
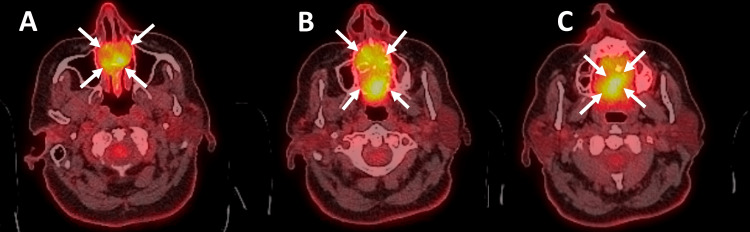
PET scan A, B, and C: PET scan showing hypermetabolic lesion in the palate without nodal uptake. (The white arrows show the tumor's limits.) PET: positron emission tomography

Positron emission tomography imaging (PET scan) showed a hypermetabolic lesion in the palate without nodal uptake (Figure 4).

These scans did not reveal any suspicious lesions elsewhere. No suspected hypermetabolic focus or submandibular and cervical lymph node involvement was found.

Two oral biopsies were undertaken but were not conclusive. The first biopsy revealed a salivary gland tumor, most likely derived from a minor salivary gland, but the specific type could not be determined with precision. The differential diagnosis included EMC or myoepithelial carcinoma. The second biopsy provided additional insights, revealing a malignant tumor originating from the accessory salivary glands. It showed a dual epithelial and predominant myoepithelial component, suggesting an EMC, with the possibility of an ACC not being definitively excluded. Based on these anatomopathological results, it was deemed necessary to establish the precise histological type of the tumor by examination of the surgical specimen. Both the biopsies and the surgical specimens were analyzed by two qualified pathologists.

**Figure 5:**
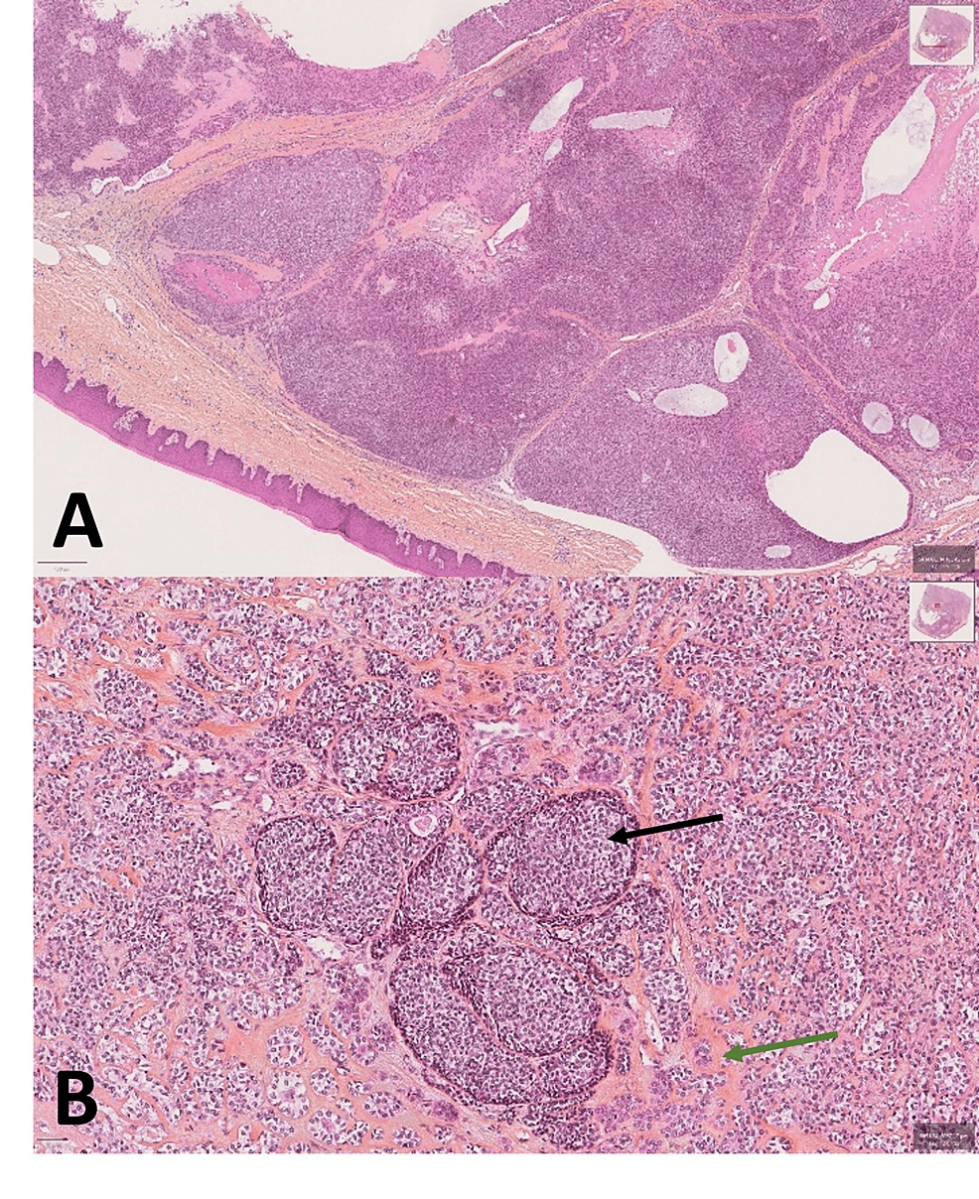
Histological section Hematoxylin and eosin (H&E) staining of the EMC tumor is characterized by the presence of both myoepithelial and epithelial components. The myoepithelial cells were predominantly observed and were surrounded by hyaline material. Magnification: x4 (A), and x10 (B). (Black arrow: Myoepithelial component. Green arrow: Epithelial component.) EMC: epithelial-myoepithelial carcinoma

On the basis of the clinical, radiological, and histological findings, this tumor was diagnosed as stage IV, with a tumor-node-metastasis staging of T4N0M0. After a multidisciplinary meeting, it was decided to perform a total maxillectomy and a reconstruction with a free fibula flap. Following the resection of the tumor, the histological analysis provided a definitive diagnosis of EMC, verifying that it was indeed a malignant tumor originating from the minor salivary glands, characterized by the presence of both myoepithelial and epithelial components. The myoepithelial cells were predominantly observed and were surrounded by hyaline material. Additionally, local progression of carcinomatous structures towards tumor necrosis was observed (Figure 5).

Immunohistochemistry analysis revealed the following findings: P63 showed diffuse and intense expression in the myoepithelial component, SOX10 displayed heterogeneous expression, while PS100, Calponin, and smooth muscle actin exhibited heterogeneous and less intense expression. The expression of cytokeratins AE1/AE3, EMA, and C-Kit (CD117) was mainly restricted to the epithelial component. C-Myb was negative, and there was no overexpression of P16 (P16 -).

Based on these clinical, radiological, and histological findings, the final diagnosis was confirmed as EMC. The patient has been followed up for a period of two years, after prosthetic rehabilitation, and no recurrence or metastasis has been observed. The patient will continue to benefit from a personalized long-term follow-up.

## Discussion

EMC is a rare tumor whose diagnosis can be clinically and histologically challenging. The absence of specific symptoms may also lead to delayed diagnosis. In this case, our patient had a longstanding lesion that had been developing for several years in the hard palate. The patient had overlooked this lesion for more than 30 years and did not seek any medical advice. Therefore, no biopsies or treatments have been provided during the long asymptomatic phase of the disease. Nasal obstruction and the onset of pain led her to seek dental consultation at the oral surgery department. However, in a case reported by Palaniappan et al., the palatal EMC was only of one-month duration and was quickly growing and becoming painful, leading to earlier diagnosis. It measured 15 mm on diagnosis. Two other palatal EMC cases were reported by Angiero et al., the first one was an asymptomatic lesion detected incidentally during a routine dental checkup with a diameter of 15 mm, and the second was an intermittent painful mass of 35×20 mm [9,10]. In our case, the mucosal swelling measured a 45-mm-long axis on diagnosis and was already crossing the medial line, with a purplish-red color and polypoid outgrowth. The tumor already involves the maxilla, the alveolar process, the nasal cavities, the nasal septum, and the maxillary sinuses. Regarding these differences, we can think that the patients of the above-mentioned case reports developed a primary malignant tumor with faster growth, while our patient might have undergone malignant transformation from a primary benign tumor, probably a palatal PA present during several years, to a secondary malignant lesion. Indeed, cases of PA transformation to EMC have been described in a few studies, with an estimated rate of malignant transformation in PA of 1.6 to 7.5% [3].

Histologically, the differential diagnosis may be confusing because of the complexities in distinguishing EMC from ACC and PA as described above. Microscopic examination shows similar biphasic composition of epithelial and myoepithelial cells in PA and EMC, which poses difficulties in the differential diagnosis. The anatomopathological analysis relies on combined HE with multi-IHC staining: EMA, p63, calponin, and the Ki-67 labeling index calculation. Vimentin and calponin are very sensitive and specific myoepithelial markers for salivary tumor myoepithelial cells that facilitate confirmation of diagnosis [3].

According to the WHO classification of 2022, the EMC is considered a low-grade tumor [8]. In a recent review, EMC arising from minor GS is reported to be characterized by about 11.8% of local recurrence rate and 94.4% of survival rate [3]. Despite the good prognosis associated with the low-grade malignancy of EMC, some reports have highlighted its rare aggressive nature.

In our case, the clinical aspect of the tumor was as an intraoral mass, like the two cases reported by Angiero et al. describing two palatal masses in a 58-year-old woman and an 83-year-old man. However, in the case reported by Palaniappan et al., the palatal EMC was presented as a partially ulcerated lesion. All these patients were free of recurrence at the follow-up appointment of 15, 13, and 12 months respectively. Our patient had been followed-up for the longest duration which is 24 months post-surgery [9,10].

As regards the management of EMCs originating from the minor salivary glands, the role of radiotherapy is not well defined. Adjuvant radiotherapy is recommended in major salivary gland tumors when the primary tumor is >4 cm in size or the surgical margins are positive [3]. However, Vazquez et al. reported 10-year survival rate differences between patients treated only by surgery and that of those treated by surgery associated with adjuvant radiotherapy (93.2% and 87.6% respectively) [1,3]. Following the recommendations of the French Rare Head and Neck Cancer Network (REFCOR), in stages I and II of low-grade tumors like EMC, radiotherapy after a complete surgical resection is not indicated [11].

It is important to make the correct histological diagnosis because this changes completely the tumor management. Indeed, the ACC which is the principal differential clinical and histological diagnosis of EMC is classified by the WHO classification as an intermediate to high-grade tumor. It carries a poorer prognosis than EMC and requires post-surgical radiotherapy in almost all stages (II-VI). Moreover, as the tumor recurrence rate is much higher, a closer follow-up is recommended. The survival at five years is more than 95 % for stage I low-grade tumors and less than 10 % for stage IV high-grade tumors [11].

Even though the efficiency of chemotherapy is not well established, some authors have suggested that chemotherapy be associated with radiotherapy when the delivered doses are high (around 65 Gy) especially when safety margins are not sufficiently extended while excision [3,12].

## Conclusions

This article reported a rare case of EMC in the maxilla treated by maxillectomy. The diagnosis of EMC remains difficult due to its rarity. There is no consensus on the optimal management of the minor salivary glands EMCs. Longer-term cohort studies would provide a better understanding of their evolution and their optimal management and avoid late diagnosis. Clear standardized recommendations should, therefore, be put in place for the care of these patients.
